# Supplementary material for: Neutrophil-specific Shp1 loss results in lethal pulmonary hemorrhage in mouse models of acute lung injury
Source: J Clin Invest. 2024 Oct 1;134(24):e183161. doi: 10.1172/JCI183161 (PMC11645157; doi:10.1172/JCI183161)
Supplement: Supplemental data [file jci-134-183161-s203.pdf]

Supplementary Data

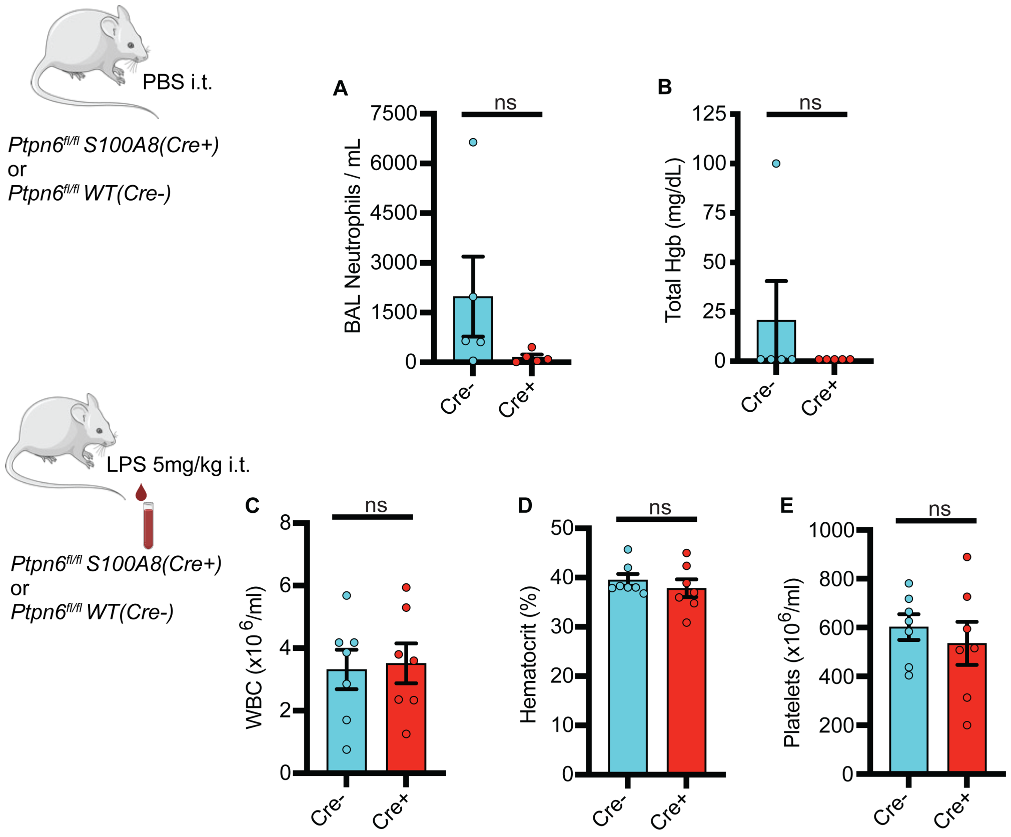

Supplemental Figure 1. Baseline BAL fluid analysis after PBS instillation and peripheral blood counts after LPS instillation. Similar (**A**) alveolar inflammation and lack of (**B**) alveolar hemorrhage in *Ptpn6*$^{fl/fl}$ and *Ptpn6*$^{fl/fl}$ *S100A8(Cre+)* 48 hours after intra-tracheal PBS instillation. Similar (**C**) blood WBCs, (**D**) hematocrit, and (**E**) blood platelet counts, 48 hours after intra-tracheal LPS instillation in *Ptpn6*$^{fl/fl}$ and *Ptpn6*$^{fl/fl}$ *S100A8(Cre+)*. Comparison by unpaired two-tailed t-tests on log_{10}-transformed data (A-E).

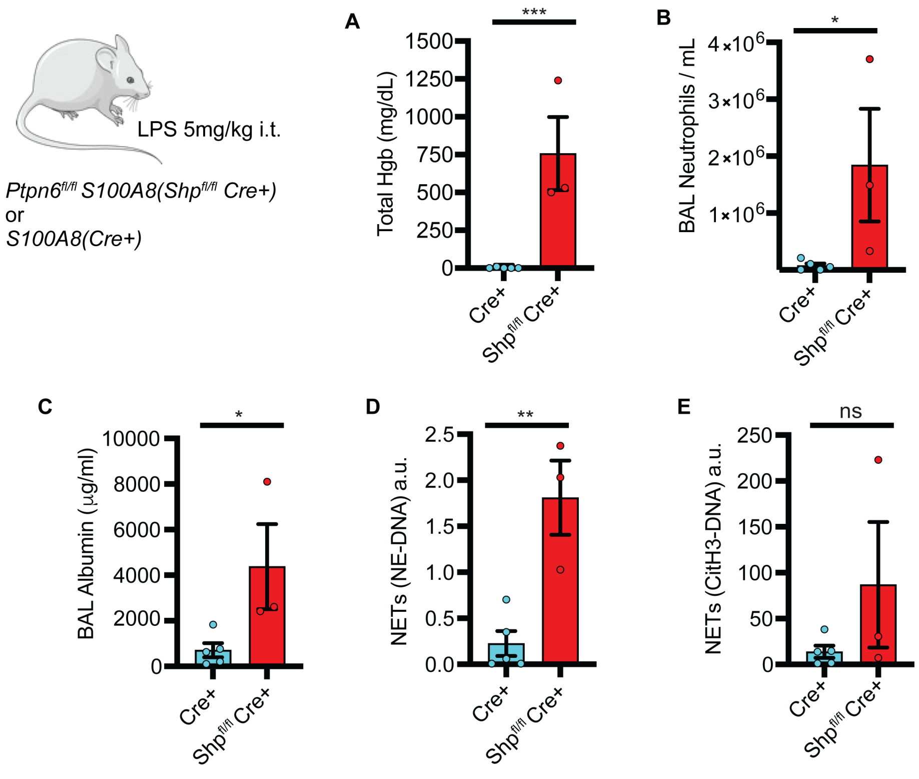

Supplemental Figure 2. LPS-induced lung hyperinflammation in *S100A8(Cre+)* vs. *Ptpn6*$^{fl/fl}$ *S100A8(Cre+)* mice. (**A**) Alveolar hemorrhage, (**B**) BAL neutrophils, (**C**) BAL albumin, (**D**) BAL NE-DNA NETs, (**E**) and BAL CitH3-DNA NETs are reduced in *S100A8(Cre+)* vs. *Ptpn6*$^{fl/fl}$ *S100A8(Cre+)* mice after LPS challenge*. ****p<0.0001, **p<0.01, *p<0.05*.

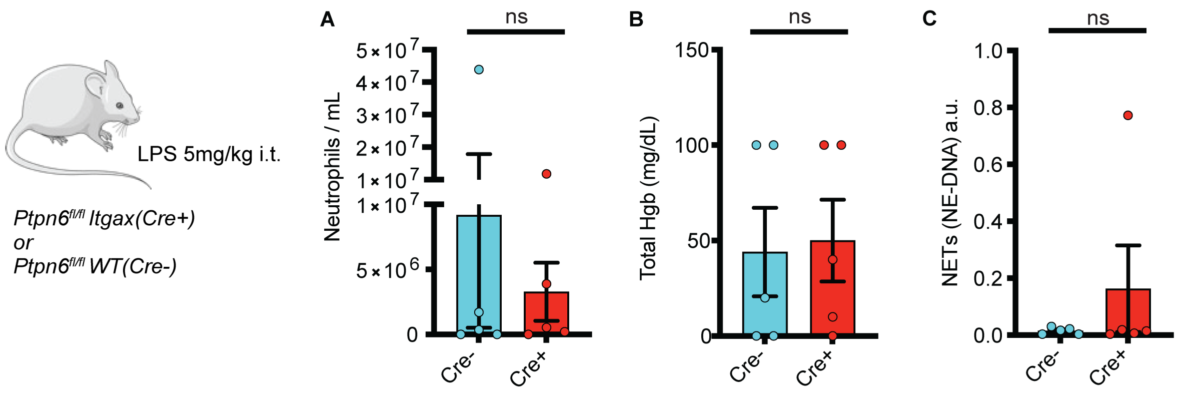

Supplemental Figure 3. Similar LPS induced lung inflammation with the loss of Shp1 in alveolar macrophages and dendritic cells. Comparable alveolar (**A**) neutrophilia, (**B**) hemorrhage and (**C**) NETs in *Ptpn6*$^{fl/fl}$ *Itgax(Cre+)* vs. *Ptpn6*$^{fl/fl}$ mice. Comparison by unpaired two-tailed t-tests on log_{10}-transformed data (A-C).

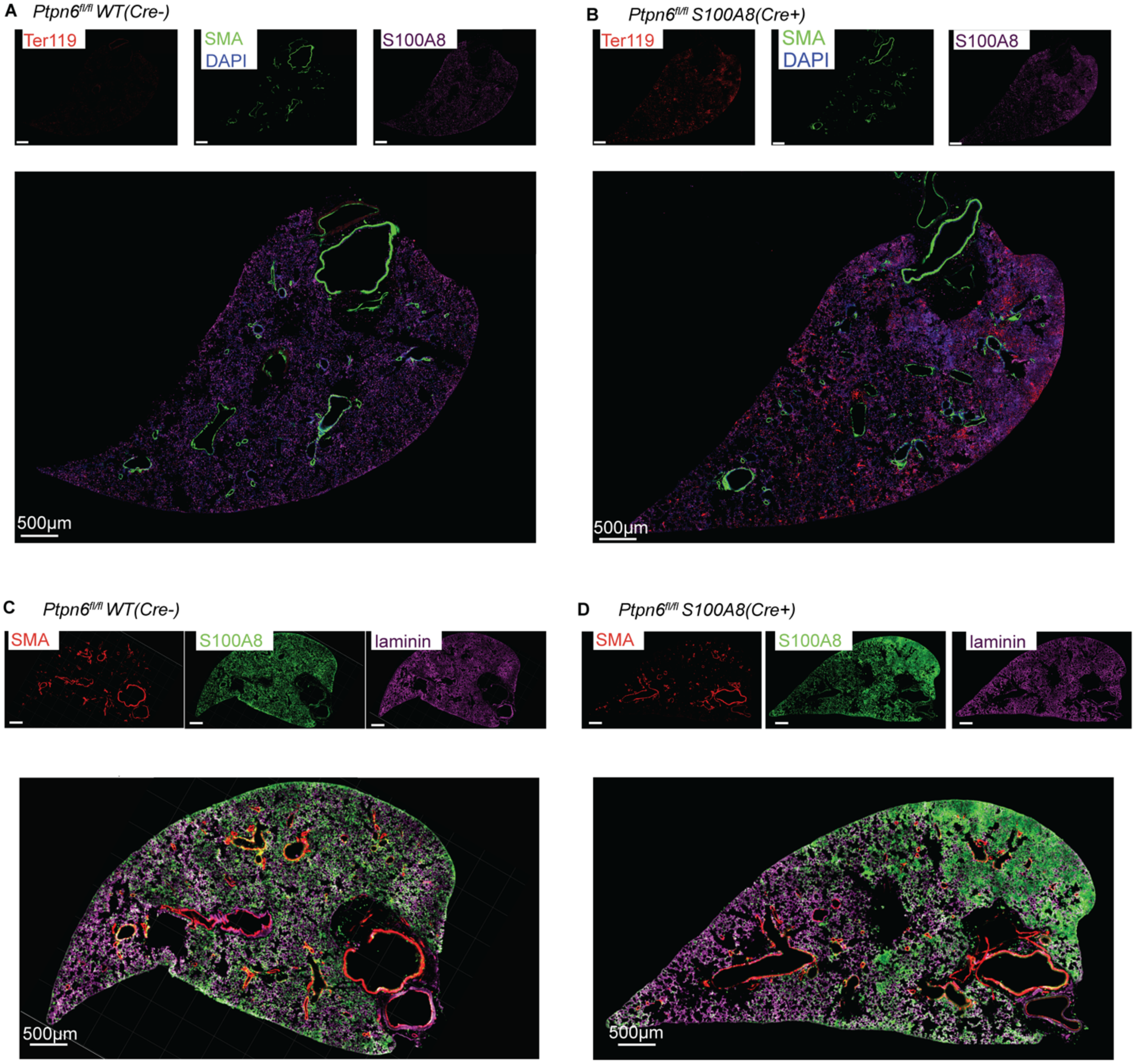

Supplemental Figure 4. Diffuse neutrophilia and parenchymal hemorrhage with the loss of neutrophil Shp1. Immunofluorescence imaging of lung tissue with staining for S100A8 (neutrophils), Ter119 (red blood cells), laminin, and smooth muscle actin (SMA) at 48 hours after LPS challenge from (**A,C**) *Ptpn6$^{fl/fl}$* and (**B,D**) *Ptpn6$^{fl/fl}$ S100A8(Cre+)* and showing increased tissue neutrophils and red blood cell stains in mice with neutrophil Shp1 deletion.

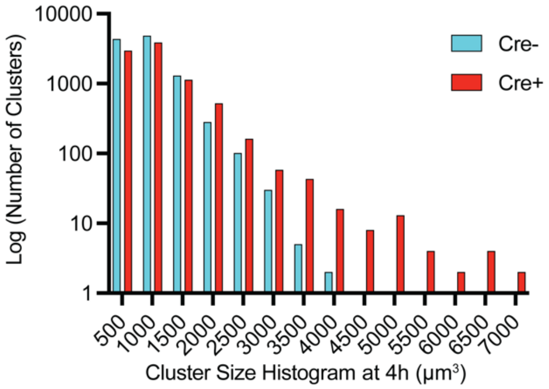

Supplemental Figure 5. Intravascular neutrophil clusters 4h after intratracheal LPS instillation. Quantification of intravital lung imaging showing increased numbers and size of intravascular neutrophil clusters 4h after LPS instillation with the loss of Shp1 in neutrophils.

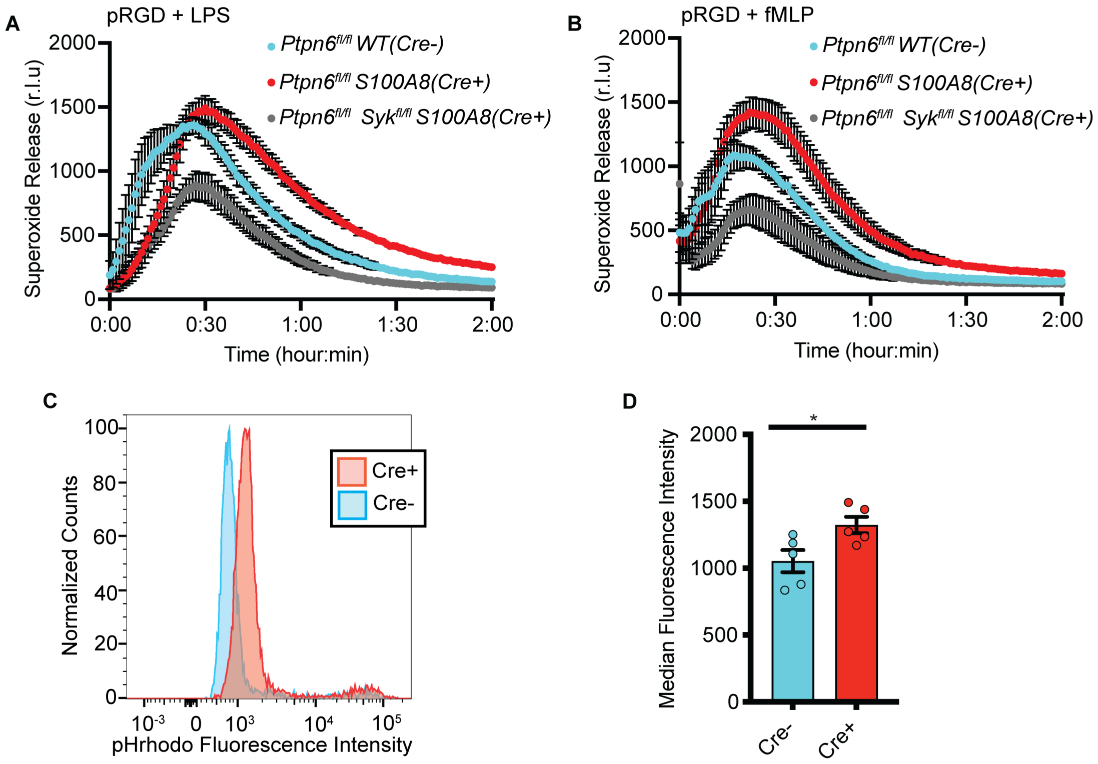

Supplemental Figure 6. Increased agonist induced reactive oxygen species (ROS) production and increased phagocytosis of pH-Rhodamine-labelled zymosan particles in Shp1 knockout neutrophils. (**A**) LPS and (**B**) fMLP induced ROS production on RGD-coated surfaces in *Ptpn6$^{fl/fl}$ S100A8(Cre+)* neutrophils is dependent on *Syk* kinase signaling. (**C**) Sample fluorescence curve with (**D**) increased mean fluorescence intensity of pH-Rhodamine zymosan particles indicating increased fluorescence in Shp1 knockout neutrophils (n=5 mice). *P* values are from unpaired two-tailed t-tests on $\log_{10}$-transformed data (F). *$*p<0.05$.*

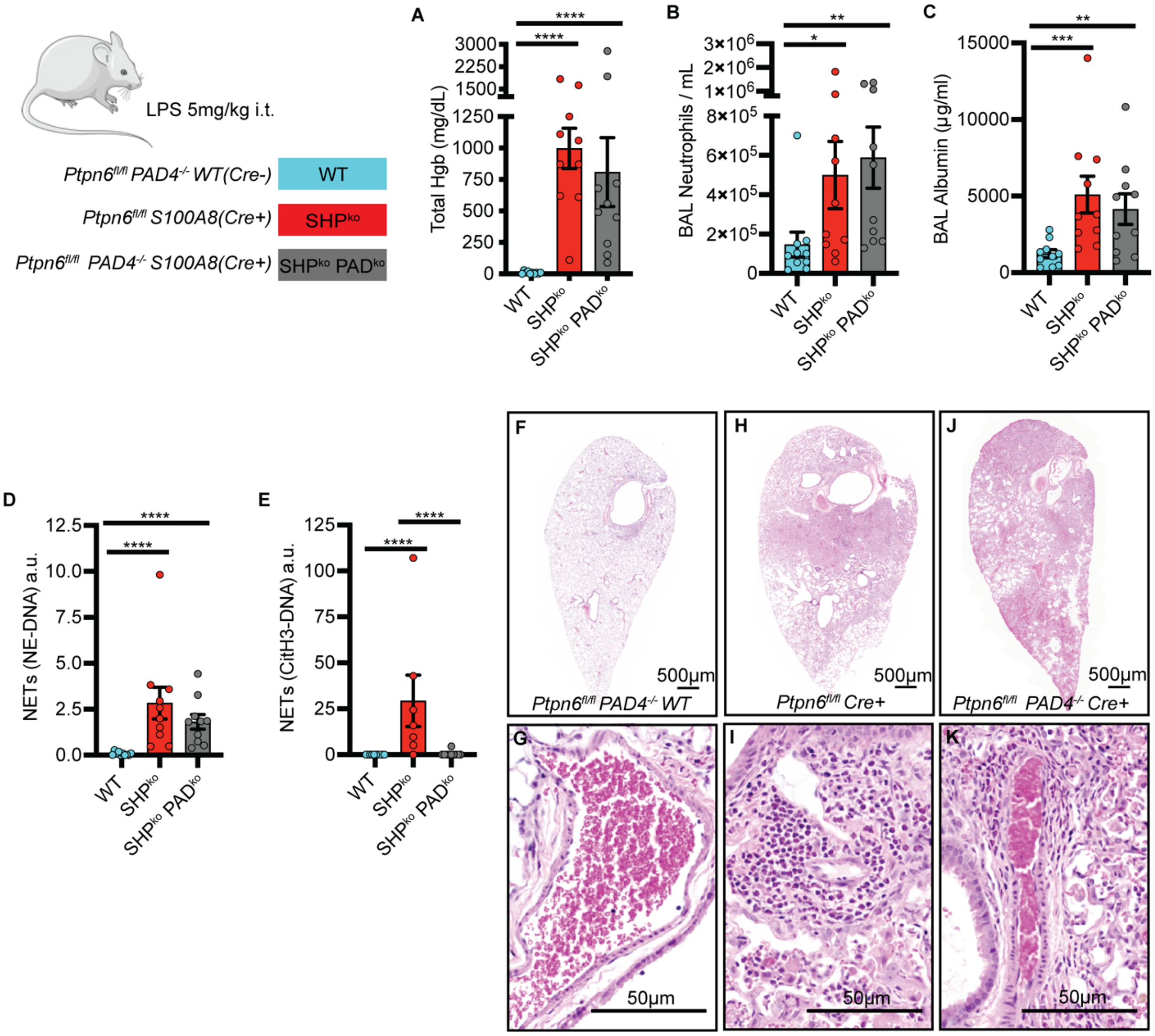

Supplemental Figure 7. LPS-induced lung hyperinflammation in neutrophil Shp1 knockouts is independent of PAD4 expression. (**A**) Alveolar hemorrhage, (**B**) BAL neutrophils, (**C**) BAL albumin, (**D**) and BAL NE-DNA NETs are unchanged in *Ptpn6*$^{fl/fl}$ *S100A8(Cre+)* vs. *Ptpn6*$^{fl/fl}$ *PAD4*$^{-/-}$ *S100A8(Cre+)* mice. (**E**) Expected reduction in CitH3-DNA complexes in *Ptpn6*$^{fl/fl}$ *PAD4*$^{-/-}$ *S100A8(Cre+)* mice. (**F-K**) H&E-stained lung tissue in (**F-G**) *Ptpn6*$^{fl/fl}$, (**H-I**) *Ptpn6*$^{fl/fl}$ *S100A8(Cre+)*, (**J-K**) *Ptpn6*$^{fl/fl}$ *PAD4*$^{-/-}$ *S100A8(Cre+)* with similar lung injury in the *Ptpn6*$^{fl/fl}$ *S100A8(Cre+)* and *Ptpn6*$^{fl/fl}$ *PAD4*$^{-/-}$ *S100A8(Cre+)* and (**I,K**) perivascular inflammation. Log_{10} transformed data were analyzed using one-way ANOVA with Tukey's test for multiple comparisons (A-E). ****$p<0.0001$, ***$p<0.001$, **$p<0.01$, *$p<0.05$. Scalebar = 50µm

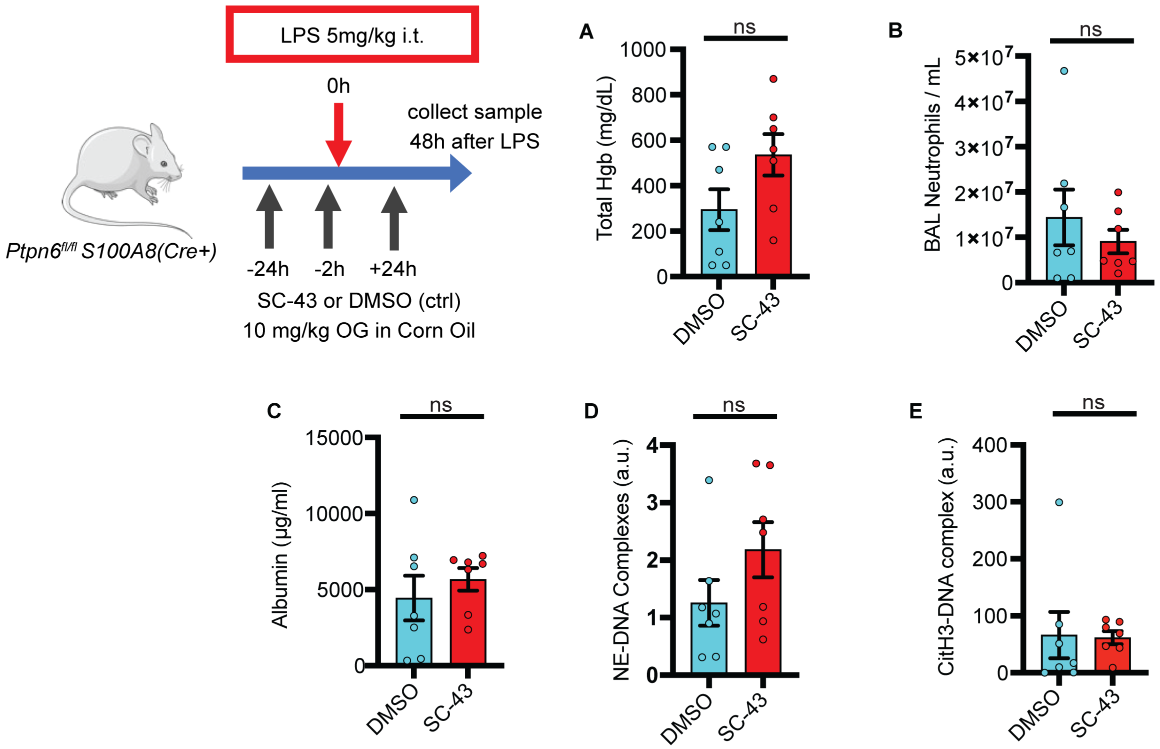

Supplemental Figure 8. Shp1 activation with SC43 does not rescue pulmonary hemorrhage and hyperinflammation associated with the loss of neutrophil Shp1. Similar (**A**) alveolar hemorrhage, (**B**) BAL neutrophils, (**C**) BAL albumin, (**D**) NE-DNA complexes, and (**E**) CitH3-DNA complexes in SC43 and DMSO (control) treated *Ptpn6$^{fl/fl}$ S100A8(Cre+)* mice after LPS challenge.

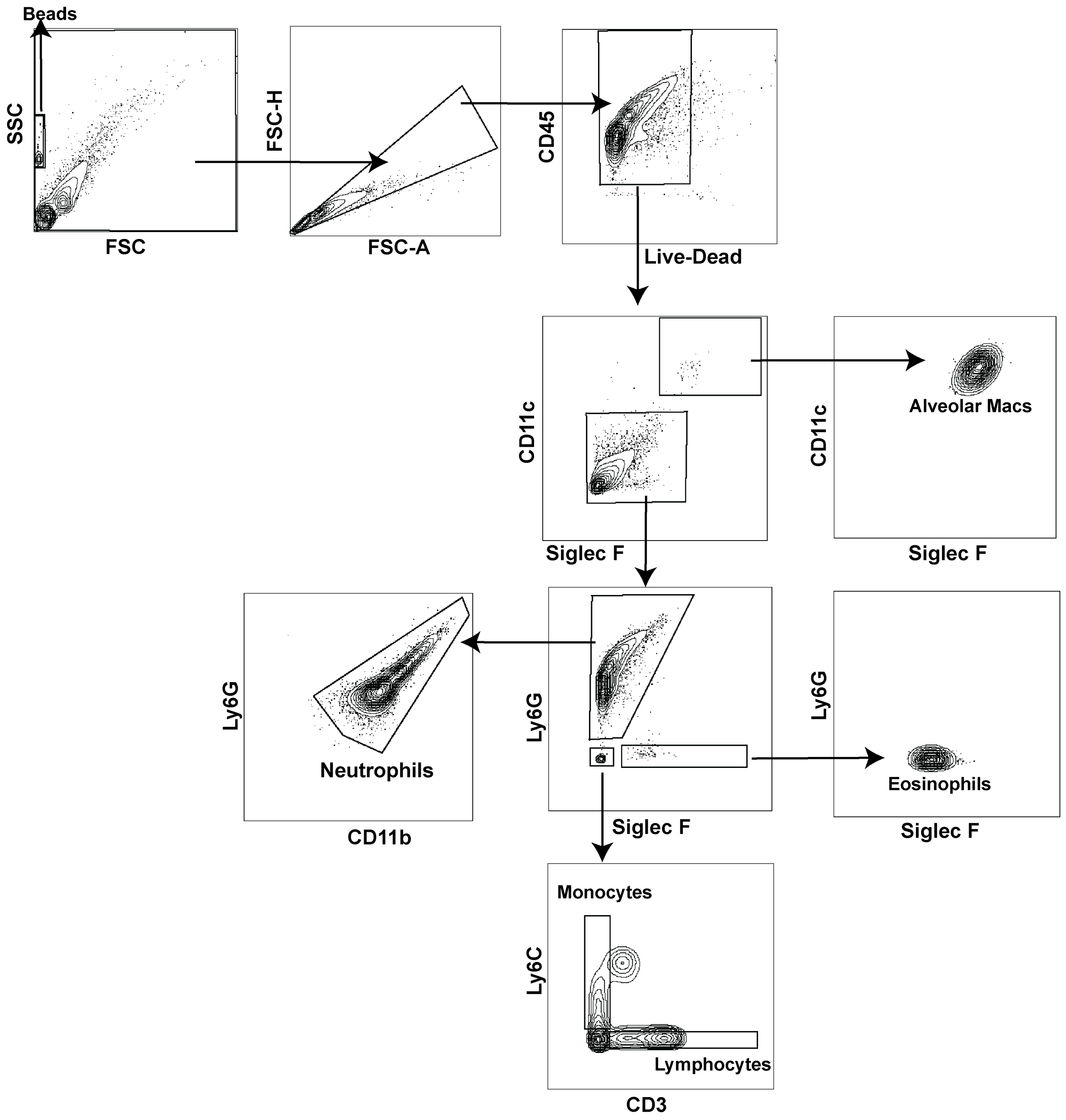

Supplemental Figure 9. Sample gating strategy for BAL collected for ALI experiments.
